# The Effectiveness of Transdiagnostic Cognitive Behavioral Therapy for Comorbid Insomnia: A Case Report

**Published:** 2018-04

**Authors:** Hoda Doos Ali Vand, Banafsheh Gharraee, Ali-Asghar Asgharnejad Farid, Mir Farhad Ghaleh Bandi, Mojtaba Habibi

**Affiliations:** 1 Department of Clinical Psychology, School of Behavioral Sciences and Mental Health (Tehran Institute of Psychiatry), Iran University of Medical Sciences, Tehran, Iran.; 2 Department of Psychiatry, Mental Health Research Center, School of Behavioral Sciences and Mental Health (Tehran Institute of Psychiatry), Iran University of Medical Sciences, Tehran, Iran.; 3 Department of Health Psychology, School of Behavioral Sciences and Mental Health (Tehran Institute of Psychiatry), Iran University of Medical Sciences, Tehran, Iran.

**Keywords:** *Anxiety*, *Depression*, *Sleep Initiation and Maintenance Disorders*, *Unified Protocol*

## Abstract

**Objective:** Similar cognitive and behavioral factors underlie and perpetuate insomnia and emotional disorders. This brief case report aims to evaluate the effectiveness of the Unified Protocol (UP), a transdiagnostic treatment designed to target emotional disorders in treating comorbid insomnia.

**Method** **:** The patient was a 32-year-old male, who met the DSM-IV-TR criteria for chronic insomnia, major depressive disorder, and generalized anxiety disorder. After 3 baseline weeks, the patient underwent 14 sessions of UP and was retested after 1-month follow-up. Consensus Sleep Diary (CSD), Insomnia Severity Index (ISI), and Pittsburgh Sleep Quality Index (PSQI) were completed during baseline, treatment, and follow-up.

**Results:** The treatment led to improvements in sleep onset latency, time awake after sleep onset, terminal awakenings, sleep quality, and insomnia severity. These gains were maintained at 1-month follow-up.

**Conclusion: **UP is effective in improving different symptoms of chronic insomnia. Controlled clinical studies with more cases are required to investigate the effects of UP in the treatment of insomnia.

There is a high comorbidity between insomnia and emotional disorders, specially anxiety and depression (1, 2). Transdiagnostic treatments that have been developed in recent years, address comorbid disorders concurrently through targeting their common and underlying factors (3). This approach assumes that disorders have more commonalities than differences, and targeting core functional relationships offer greater benefits in comparison to disorder-specific treatments (4). Unified Protocol (UP) is a transdiagnostic treatment that applies common CBT techniques in a modular format to target emotion dysregulation (5).

Common cognitive and behavioral factors (e.g., dysfunctional cognitions, attentional biases, and safety behaviors) may underlie and perpetuate both insomnia and emotional disorders (6). In addition, emotion dysregulation (7, 8), neuroticism (9, 10), and anxiety sensitivity (11-13), have been identified as significant factors contributing to insomnia and emotional disorders. 

Although previous studies have provided support for the effects of transdiagnostic treatment for emotional disorders (14-17), the impact of the UP for comorbid conditions such as insomnia requires further investigation. 

Thus, the present case study aimed at investigating the effects of UP, a transdiagnostic treatment developed to target emotional disorders, on insomnia symptoms of a patient, who complained of both insomnia and emotional disorders.

**Patient Information**

The following is a case report to illustrate the process of treatment with UP in the context of insomnia with an Iranian patient. Some of the information is changed to protect the patient’s anonymity. Patient’s informed consent was obtained prior to the initiation of the study. 

The patient, S.A., was a 32-year-old single male with a bachelor’s degree in electrical engineering, who was working part-time in a private company. He was referred to the sleep clinic by a psychiatrist for his sleep problems, and was, then, referred by a sleep clinician for psychotherapy. The patient was evaluated using Structured Clinical Interview for DSM-IV Disorders–I (SCID-I) (18, 19) and Global Sleep Assessment Questionnaire (GASQ) (20) by the first author (a clinical psychologist. To verify the presence of quantitative (21) and research diagnostic criteria for insomnia (22) the patient was asked to complete Consensus Sleep Diary (CSD) (23) for two weeks. As a result of sleep problems, he felt fatigued, sleepy, and irritable during the day and did not have enough energy and concentration for his daily tasks. He reported that his sleep problems gradually developed over the course of 4 years and were characterized by difficulties with sleep initiation, sleep maintenance, and early morning awakenings. He had a past history of recurrent depression and reported that the last episode began one year ago due to career problems. Moreover, he reported severe anxiety in relation to his work, family, and daily activities that was initiated during the past two years. He had participated in 10 sessions of supportive psychotherapy for previous episodes of depression, which was effective in reducing his depression symptoms. The total evaluation confirmed the diagnosis of chronic insomnia, moderate major depressive disorder, and generalized anxiety disorder. He was asked to complete Sleep Diary, Insomnia Severity Index (ISI) (24, 25), and Pittsburgh Sleep Quality Index (PSQI) (26, 27) during the baseline, treatment and at 1-month follow-up. 

**Therapeutic Intervention**

The treatment was conducted according to the Unified Protocol for Transdiagnostic Treatment of Emotional Disorders (5) and consisted of 14 weekly sessions. Adapting UP concepts for insomnia was based on a book chapter describing application of UP for a patient with insomnia disorder (28). Each session lasted about 60 minutes and addressed UP concepts regarding insomnia and emotional disorder symptoms. As the utilization of the UP concepts for emotional problems had previously been described (29), this case report is only dedicated to applying UP concepts for insomnia symptoms. 

Module 1 (Session 1): “Motivation Enhancement for Treatment Engagement”

To increase patient’s motivation for change, the advantages and disadvantages of “changing versus staying the same” was discussed. He mentioned “being more precise and productive at work, having more energy to deal with his friends, and having more concentration to do home tasks” as advantages of changing and “difficulty of changing habitual behaviors and the time needed to practice new behaviors” as disadvantages of change. Then, to increase patient’s self-efficacy, manageable goals and steps to achieve these goals were determined collaboratively. 

Module 2 (Session 2): “Psychoeducation and Tracking Emotional Experiences”

The three-component model was introduced and the interaction between thoughts, physical sensations, and behaviors in relation to insomnia was discussed. He reported, “I stay awake all night, so I won’t be able to work, and I will be fatigued and irritable in the next day.” as major thoughts at bedtime. He stated that he experienced dizziness, sweating, and increased heart rate in response to these thoughts. In addition, he was engaged in many disruptive behaviors such as staying in bed while awake, studying books in bed, and going to bed much earlier to compensate for sleep loss. The short- and long-term consequences of the behaviors (temporal rest versus maintaining his sleep problem) were discussed. He was able to understand that these behaviors made him feel better in the short- term, but the sleep difficulties maintained in the long-term. As targeting sleep problems before emotion regulation problems may be helpful for skill acquisition and enhancing treatment outcomes (30), we tried to target sleep problems at early stages of treatment. In this regard, reducing the time spent in bed was considered as an alternative way. Considering the fact that sudden reduction in the time spent in bed was difficult for the patient, so the reduction was introduced gradually. He agreed to alter his sleep routine by going to bed 20 minutes later each night and leaving bed when incapable of falling asleep.

Module 3 (Sessions 3-4): “Emotion Awareness Training”

A review of sleep diary data revealed a minor improvement in sleep efficiency. By the third session, the concept of “nonjudgmental present- focused emotion awareness” was introduced. He learned how to observe thoughts, feelings, and behaviors in an objective way and use breath as a cue to remind him to focus on the present moment. In this regard, acceptance of the current sleep state without trying to control or manage sleep was discussed. The patient completed a formal mindfulness exercise in the session. He found this exercise as an interesting and somewhat difficult experience and agreed to practice the mindfulness exercise once a day during the next week. In the fourth session, he stated that although nonjudgmental observation of emotional experiences was difficult at first, he was going to be better in it by practicing it. To help the patient practice the mindfulness exercise in the context of emotional experiences, emotional awareness was practiced by listening to a meaningful music and identifying emotions and reactions to them. Moreover, He was instructed to promote an observer stance during the night when unable to fall asleep and during the day when feeling fatigued, irritable, and sleepy. He agreed to practice “mood induction” and “anchoring in the present” exercise during the day in the next week. In addition, he was prescribed to continue restricting time spend in bed.

Module 4 (Sessions 5-6): “Cognitive Appraisal and Reappraisal”

A review of sleep diary data showed that sleep efficiency increased compared to the previous module. He reported that staying in touch with the present moment in an acceptable way reduced his rumination and worry regarding sleep. He became aware that nighttime and daytime symptoms of insomnia were not problematic, but engaging in behaviors to avoid these states was the problem. By the fifth session, the patient was instructed to identify negative automatic thoughts (NATs) and common cognitive errors contributing to his sleep problem. He reported the negative thoughts he usually experienced when lying in bed, such as “I won’t get enough sleep tonight; I will be irritable and tired tomorrow; I will do poorly at work.” The interaction between thoughts and emotions was discussed and he agreed to identify and record appraisals and related emotions during the next week. In the next session, “identifying and evaluating appraisals form” was reviewed and the thought “I would have a poor night sleep” was considered more deeply using “downward arrow technique” and underlying appraisals were identified as follow: “I will make a mistake at work and I will get fired.” This thought was challenged at first by identifying “thinking traps”. He was explained that this is a kind of catastrophizing and probability overestimation, and he was instructed to alternate this thought using cognitive reappraisal strategies such as decatastrophizing and countering probability overestimation. Learning to generate alternative appraisals, he replaced the thought with a more flexible one as “Even if I cannot sleep well, I can still do my tasks and the probability of getting fired is too low. Even if this happens, my past experiences show that I have the ability to cope with it.” He was encouraged to identify the core appraisals and use the reappraisal process to generate alternative appraisals. 

Module 5 (Session 7): “Emotion Avoidance and Emotion-Driven Behaviors”

He reported that cognitive reappraisal had helped him reduce his anxiety during bed time. Various kinds of emotion avoidance and maladaptive emotion-driven behaviors (EDBs) and the role of these behavioral components in emotional experiences were introduced. The patient identified a number of his dysfunctional behaviors, such as planning to go to bed earlier, napping, reducing social conversations and daily activities, and trying to save energy in anyway. He confirmed that although these behaviors may be useful in the short run by reducing his anxiety regarding sleep, they have not been helpful in the long run. He agreed to change these maladaptive behaviors by accepting a pattern of approach rather than avoiding and engaging in alternative behaviors. 

Module 6 (Session 8): “Awareness and Tolerance of Physical Sensations”

He noted that engaging in alternative behaviors and particularly having a normal level of daily activities, rather than reducing them due to poor sleep at the previous week, made him feel better. The role of physical sensations in emotional experiences and the interaction between these sensations and cognitions and behaviors was discussed. He confirmed that experiencing physical sensations play a significant role in exacerbating his anxiety, while lying in bed incapable of falling asleep. He identified increased heart rate as a major internal physical sensation associated with his anxiety about sleep. To increase tolerance of this physical sensation, running in place was conducted as a symptom induction test to elicit most similar physical sensations. After 3 trials, the intensity of symptoms and the level of distress were reduced from 8 to 5. He agreed to engage in more symptom induction exercises each day during the next week.

Module 7 (Sessions 9-13): “Interoceptive and Situation-based Emotion Exposures”

Upon coming back to the clinic for session nine, Mr. S.A. reported that he was practicing hyperventilation as a symptom induction test several times, but his distress never reduced. A more detailed assessment of the situation revealed that he was not fully engaged in the exercise. This was conceptualized as avoidance and after modifying anxious beliefs about this situation; he was instructed to practice with the intensity necessary to fully produce physical sensations. The rationale of emotion exposures was introduced and an avoidance hierarchy in relation to sleep situations was developed. The situations were ranked according to their difficulty. As a first exposure, he agreed to sleep without any earplugs. As a second exposure, he agreed to stop checking the clock during the night. As a third exposure, he agreed to engage in a normal level of social activities rather than reducing them. Another exposure was going to bed at a normal bedtime, stopping postponing tasks to the next days, and stopping cancelling appointments. In addition to situational exposures, interoceptive exposures (e.g., hyperventilation), which were instructed in the previous module, were continued. 

Module 8 (Session 14): “Relapse Prevention”

Key treatment skills were reviewed, and treatment progress was evaluated. By reviewing sleep questionnaires on a weekly basis, he confirmed that his insomnia severity had reduced gradually. He reported that he was more satisfied with his sleep quality, and his total wake time had reduced significantly. He confirmed some progress in “present focus emotion awareness”, “negative thoughts”, “symptom and activity exercises”, and “avoidance and emotion-driven behaviors”. However, he believed that there are other activities and situations for which he should develop a practice plan. Fluctuation of sleep problems (experiencing occasional poor nights of sleep) was discussed as a normal condition and he was instructed to see these situations as an opportunity to practice learned skills more and get back on track.

**Outcomes**

As observed in Figures 1 and 2, sleep onset latency (SOL), wake after sleep onset (WASO), and terminal waking after sleep onset (TWASO) decreased from pre- to post-treatment and follow-up. This was accompanied by a reduction in ISI and PSQI scores. His score on ISI showed that he had reached a subclinical level of insomnia (<15 on ISI) at post-treatment and absence of insomnia (<8 on ISI) at follow-up. Moreover, SOL and WASO reached below the clinical cut-off of 30 minutes at post-treatment and follow-up. 

## Discussion

It was illustrated by a brief case report how UP modules can be used to reduce insomnia symptoms. UP techniques may be helpful in the treatment of chronic insomnia comorbid with emotional disorders. Regarding the gradual decrease of insomnia symptoms during treatment, all modules of UP may be important in reducing insomnia symptoms. Module 1 (by increasing readiness for change), module 2 (by helping clients to develop greater awareness of sleep emotional experiences), module 3 (by allowing clients to objectively observe daytime and nighttime symptoms of insomnia), module 4 (by modifying sleep-related dysfunctional cognitions), module 5 (by identifying maladaptive avoidances and EDBs perpetuating insomnia), module 6 (by increasing awareness and toleration of physical sensations during night and day), module 7 (encouraging clients to approach sleep-related avoided situations),and module 8 (promoting continued progress) may affect sleep problems. In general, UP may result in important changes in co-occurring diagnoses by targeting core underlying factors (17).

**Figure 1:**
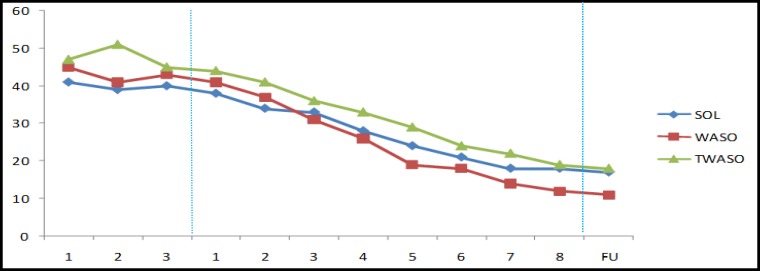
Results of SOL, WASO & TWASO During the Study

**Figure 2:**
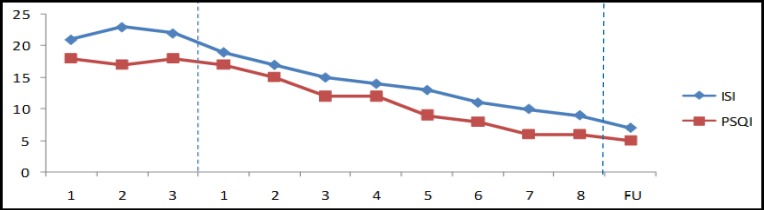
Results of ISI and PSQI Scores During the Study

## Conclusion

This case report represents one of the first attempts in the literature to adapt UP concepts to treat insomnia. UP has the potential to target symptoms of both insomnia and emotional disorders in each session. It can help therapists to target insomnia symptoms concurrent with emotional symptoms at the early stages of treatment. Well-designed research with larger samples is required to examine the efficacy of UP in individuals with comorbid insomnia and emotional disorders. We are currently in the process of gathering data on the effects of UP in a comorbid insomniac sample using a single case experimental design.

## Limitation

Our results were limited to one patient with a short follow-up period. However, this study can be served as a beginning point for further research. 
